# Measuring waning protection from seasonal influenza vaccination during nine influenza seasons, Ontario, Canada, 2010/11 to 2018/19

**DOI:** 10.2807/1560-7917.ES.2024.29.8.2300239

**Published:** 2024-02-22

**Authors:** Hannah Chung, Michael A Campitelli, Sarah A Buchan, Aaron Campigotto, Natasha S Crowcroft, Jonathan B Gubbay, James KH Jung, Timothy Karnauchow, Kevin Katz, Allison J McGeer, J Dayre McNally, David C Richardson, Susan E Richardson, Laura C Rosella, Margaret L Russell, Kevin L Schwartz, Andrew Simor, Marek Smieja, Maria E Sundaram, Bryna F Warshawsky, George Zahariadis, Jeffrey C Kwong, on behalf of the Canadian Immunization Research Network (CIRN) Provincial Collaborative Network (PCN) Investigators

**Affiliations:** 1 ICES, Toronto, Canada; 2 Dalla Lana School of Public Health, University of Toronto, Toronto, Canada; 3 Public Health Ontario, Toronto, Canada; 4 Hospital for Sick Children, Toronto, Canada; 5 London Health Sciences Centre, London, Canada; 6 Centre for Vaccine Preventable Diseases, University of Toronto, Toronto, Canada; 7 Department of Laboratory Medicine and Pathobiology, University of Toronto, Toronto, Canada; 8 Children’s Hospital of Eastern Ontario, Ottawa, Canada; 9 Department of Pathology and Laboratory Medicine, University of Ottawa, Ottawa, Canada; 10 North York General Hospital, Toronto, Canada; 11 Sinai Health System, Toronto, Canada; 12 William Osler Health System, Brampton, Canada; 13 Cumming School of Medicine, University of Calgary, Calgary, Canada; 14 Sunnybrook Health Sciences Centre, Toronto, Canada; 15 McMaster University, Hamilton, Canada; 16 Center for Clinical Epidemiology and Population Health, Marshfield Clinic Research Institute, Marshfield, United States; 17 Western University, London, Canada; 18 Newfoundland and Labrador Public Health Laboratory, St. John’s, Canada; 19 Department of Family and Community Medicine, University of Toronto, Toronto, Canada; 20 University Health Network, Toronto, Canada; 21 https://cirnetwork.ca/network/provincial-collaborative-network

**Keywords:** Influenza vaccine, vaccine effectiveness, waning immunity

## Abstract

**Background:**

Waning immunity from seasonal influenza vaccination can cause suboptimal protection during peak influenza activity. However, vaccine effectiveness studies assessing waning immunity using vaccinated and unvaccinated individuals are subject to biases.

**Aim:**

We examined the association between time since vaccination and laboratory-confirmed influenza to assess the change in influenza vaccine protection over time.

**Methods:**

Using linked laboratory and health administrative databases in Ontario, Canada, we identified community-dwelling individuals aged ≥ 6 months who received an influenza vaccine before being tested for influenza by RT-PCR during the 2010/11 to 2018/19 influenza seasons. We estimated the adjusted odds ratio (aOR) for laboratory-confirmed influenza by time since vaccination (categorised into intervals) and for every 28 days.

**Results:**

There were 53,065 individuals who were vaccinated before testing for influenza, with 10,264 (19%) influenza-positive cases. The odds of influenza increased from 1.05 (95% CI: 0.91–1.22) at 42–69 days after vaccination and peaked at 1.27 (95% CI: 1.04–1.55) at 126–153 days when compared with the reference interval (14–41 days). This corresponded to 1.09-times increased odds of influenza every 28 days (aOR = 1.09; 95% CI: 1.04–1.15). Individuals aged 18–64 years showed the greatest decline in protection against influenza A(H1N1) (aOR_per 28 days_ = 1.26; 95% CI: 0.97–1.64), whereas for individuals aged ≥ 65 years, it was against influenza A(H3N2) (aOR_per 28 days_ = 1.20; 95% CI: 1.08–1.33). We did not observe evidence of waning vaccine protection for individuals aged < 18 years.

**Conclusions:**

Influenza vaccine protection wanes during an influenza season. Understanding the optimal timing of vaccination could ensure robust protection during seasonal influenza activity.

**Key public health message**

**What did you want to address in this study and why?**

Protection from seasonal influenza vaccination decreases during an influenza season, possibly because of declining antibody levels. However, influenza activity and the timing of influenza vaccination availability varies between regions. Our aim was to understand how influenza vaccine protection changes over the course of an influenza season. This information would be useful to determine the optimal timing of the annual influenza vaccination.

**What have we learnt from this study?**

The extent to which influenza vaccine protection declines during an influenza season may differ between different age groups and different influenza types/subtypes.

**What are the implications of your findings for public health?**

Evidence of waning influenza vaccine protection can inform policy recommendations on the timing of the annual influenza vaccination. This can ensure that the population has sustained protection throughout the season, particularly during peak influenza activity.

## Introduction

The timing of annual influenza vaccination is important because antibody levels decline over time [1-3], potentially leading to suboptimal protection during peak influenza activity. In Canada, influenza vaccines are available starting in October, but influenza activity typically does not start until late November/early December and peaks in January/February. Vaccinating closer to the start of increasing activity may optimise protection, but delaying vaccination could inadvertently reduce vaccine coverage [4] and result in infections before vaccination.

Test-negative design studies have demonstrated that influenza vaccine effectiveness (VE) decreases approximately 33 percentage points ≥ 3 months after vaccination [5]. However, comparisons between vaccinated and unvaccinated populations can show spurious waning VE because of the depletion-of-susceptibles bias, where the proportion at risk of infection decreases faster among those unvaccinated than vaccinated [6,7]. Vaccinee-only studies also demonstrated that influenza vaccine protection decreases during a season. Studies from Singapore and the United States (US) found that the odds of influenza infection increased over time since influenza vaccination [8,9].

Evidence on waning influenza vaccine protection can inform recommendations on the timing of vaccination, and since influenza activity (e.g. influenza season onset, length, peak periods), vaccine availability and policies vary between regions, understanding waning vaccine protection in a local context is important. Our study objective was to evaluate influenza vaccine protection over time since vaccination during the 2010/11 to 2018/19 influenza seasons in Ontario, Canada, for all ages and any influenza types/subtypes, and stratified by age group, by age group and influenza type/subtype and by influenza season, in order to confirm and build on findings from previous studies.

## Methods

### Study population, setting, and design

We linked datasets containing respiratory virus tests performed by public health and hospital laboratories to provincial health administrative databases [10]. These datasets were linked using unique encoded identifiers and analysed at ICES. Starting from the 2016/17 season, we included data contained in the Ontario Laboratories Information System. We restricted the analysis to community-dwelling individuals aged ≥ 6 months in Ontario, Canada who were vaccinated against influenza and tested for influenza within the same influenza season (in any season between 2010/11 and 2018/19).

We determined the odds of different time-since-vaccination intervals between those who tested positive for influenza (cases) and those who tested negative (controls).

### Data sources and definitions

#### Laboratory data

We included laboratory results for respiratory specimens tested for influenza by reverse transcription PCR (RT-PCR) and defined each unique specimen collection day as a testing episode. We defined each influenza season as starting on 1 October and ending on 31 March of the following year (e.g. the 2010/11 influenza season was from 1 October 2010 to 31 March 2011) to ensure consistent observation time. For cases, we included their first testing episode that was positive for influenza after vaccination to emulate the primary outcome of a time-to-event analysis if waning influenza vaccine protection was estimated using a cohort study design. For individuals without any positive testing episodes (i.e. controls), we included their first negative testing episode after vaccination. Both vaccination and the selected testing episode had to be within the same influenza season. We excluded individuals who tested positive for influenza by any method before vaccination within the same season. The specimen collection date was the index date.

#### Influenza vaccination

We ascertained physician- and pharmacist-administered influenza vaccinations from billing claims recorded in the Ontario Health Insurance Plan (OHIP) and the Ontario Drug Benefit (ODB) databases. The claim date was assumed to be the vaccination date. *Current* season vaccination was defined as a claim after 1 October of the index season, whereas *prior* season vaccination was a claim between 1 October and 31 August in the season before the index season.

Individuals were fully vaccinated against influenza if they met age-based recommendations as of the index date (i.e. one dose for those ≥ 9 years; one dose for children aged 6 months to < 9 years who were vaccinated in any previous season; two doses ≥ 4 weeks apart for children aged 6 months to < 9 years who were first time influenza vaccine recipients) [11]. We excluded individuals: (i) who had more than one influenza vaccine in the same season (except first time recipients aged 6 months to < 9 years) since additional doses could impact antibody levels; (ii) who only had the influenza vaccine tracking code billed in OHIP, which does not provide the date of vaccine receipt; (iii) who were first time recipients aged 6 months to < 9 years but only received one dose; (iv) who met the fully vaccinated definition < 14 days before the index date (since sufficient immune responses can take up to 14 days after vaccination to develop); and (v) who received their influenza vaccine in the month of September, as these billing claims were likely to be data entry errors. Finally, to mitigate the impact of the depletion-of-susceptibles bias, which can also affect vaccinee-only studies [6,7], we excluded individuals who were vaccinated after the start of influenza activity, which was the first week in each season where the weekly influenza testing positivity was > 5%. The start and end dates of influenza activity in Ontario and other influenza season characteristics are provided in Supplementary Table S1.

Time since vaccination was calculated as the number of days between the fully vaccinated and index dates, which we parameterised into intervals (14–41, 42–69, 70–97, 98–125, 126–153 and ≥ 154 days) and used this categorical variable as the main exposure in the analyses. We also divided time since vaccination by 28 days and used the continuous variable as an alternate exposure. In Ontario, standard-dose trivalent influenza vaccine (TIV) was administered throughout the study period, whereas standard-dose quadrivalent influenza vaccine (QIV), including live attenuated influenza vaccines (LAIV) for individuals aged 2–17 years, was only available starting from the 2015/16 season.

#### Covariates

We obtained age (categorised as 6 months to 17 years, 18–49, 50–64, 65–74, 75–84 and ≥ 85 years), sex (male/female), neighbourhood income quintile, rurality and Public Health Unit (PHU) region from the Registered Persons Database as of index date. We also determined healthcare utilisation (number of hospitalisations in the past 3 years, number of outpatient visits and prescription medications in the past year and receipt of home care services in the past year before index date) using the Canadian Institute for Health Information’s Discharge Abstract Database, Home Care Database, OHIP and ODB databases.

We identified comorbidities that increase the risk of severe disease due to influenza. Validated algorithms and diagnostic/procedure codes used to identify these comorbidities from health administrative databases have been described elsewhere [10,12].

### Statistical analysis

We conducted model-building exercises to identify the characteristics that needed to be accounted for in the main models that estimated the association between time-since-vaccination intervals and laboratory-confirmed influenza. We identified characteristics that were possible confounders. Candidate characteristics included age group, sex, neighbourhood income quintile, rurality, healthcare utilisation, any comorbidity and prior season influenza vaccination. These characteristics were selected a priori because of their known associations with influenza vaccine receipt. To assess the association between each characteristic and time-since-vaccination intervals, which was used as a categorical outcome in this relationship, we used multinomial logistic regression. Separately, we assessed the association between each characteristic and laboratory-confirmed influenza using logistic regression. Characteristics that were significant (type 3 analysis p *<* 0.05) in univariate analyses in both models were then assessed to determine whether their inclusion in the main model changed the magnitude for any time-since-vaccination interval beta-coefficient by > 10%. We also performed a likelihood ratio test to confirm the collective importance of the covariates as confounders. 

We decided a priori to account for PHU region and week of influenza test (i.e. index week) in our main model since influenza activity varies by calendar time and geography. Also, influenza vaccine availability is coordinated by PHUs, which could affect when individuals in a given region were vaccinated. To confirm its importance to include in the models, we compared Akaike’s information criterion (AIC) values between models that conditioned and did not condition for Public Health Unit region and index week.

To assess waning vaccine protection, we calculated adjusted odds ratios (aORs) by comparing the odds of laboratory-confirmed influenza for each time-since-vaccination interval with the reference interval (14–41 days) using conditional logistic regression, conditioning on PHU region and index week and adjusting for the identified confounders. We tested whether there was a trend with increasing time-since-vaccination interval categories and the odds of the outcome using a Cochran–Armitage trend test [13].

We also determined the odds of laboratory-confirmed influenza for every 28 days on a continuous basis (by dividing the number of days since vaccination by 28). We tested for linearity between this continuous variable and the logit of the outcome by creating restricted cubic splines and assessing whether its regression coefficients were collectively different than zero [14,15].

We assessed vaccine protection over time using both representations of time since vaccination: (i) against any influenza and separately against each influenza type/subtype (A(H1N1), A(H3N2) and B), and (ii) by age group (< 18, 18–64 and ≥ 65 years) by combining data from the 2010/11 to 2018/19 seasons; and (iii) separately for the predominant subtype(s) for each influenza season.

We conducted a sensitivity analysis to determine whether changing the influenza season definition and length to only include vaccinated individuals tested for influenza during periods of influenza activity (i.e. weeks with > 5% influenza positivity), would impact our overall and season-specific results. In addition, we conducted two negative tracer analyses to determine the specificity of our results. To assess for residual confounding, we conducted a negative tracer *outcome* analysis to evaluate time since influenza vaccination and laboratory-confirmed infection with respiratory syncytial virus (RSV), against which influenza vaccination is not expected to provide protection. We also performed a negative tracer *exposure* analysis using unvaccinated individuals who were also tested for influenza to determine whether the association between time since vaccination and influenza was due to the inherent epidemic waves (e.g. individuals vaccinated at shorter intervals (e.g. 14–41 days) had specimens collected before peak influenza activity, i.e. October, November). We first determined the distribution of the lengths of the time-since-vaccination intervals for each age group, season and index month stratum among vaccinated individuals. We then randomly assigned an interval length to each unvaccinated individual from the same stratum (while maintaining the same interval length distribution as the vaccinated group). We derived a pseudo-vaccination date by subtracting the interval length from the influenza testing date. If there was an association between time since pseudo-vaccination intervals and influenza (i.e. if aOR > 1.0), then the association between time since vaccination and influenza among vaccinated individuals could be impacted by biases.

All analyses were conducted using SAS Version 9.4 (SAS Institute Inc., Cary, US). All tests were two-sided and aOR 95% confidence intervals (CI) including 1.0 were deemed insignificant. Cochran–Armitage trend tests with p < 0.05 implied that there was a trend between time-since-vaccination intervals and laboratory-confirmed influenza. Tests for linearity with p ≥ 0.05 (i.e. accepting the null hypothesis that the restricted cubic spline coefficients are zero) implied that there was a linear association between the 28-day time-since-vaccination continuous variable and the logit of the outcome.

## Results

After combining data from nine influenza seasons, there were 53,065 individuals tested for influenza after vaccination during the same influenza season, with 10,264 (19%) testing positive. Characteristics identified as confounders in the relationship between time-since-vaccination groups and laboratory-confirmed influenza included age group, presence of comorbidities, number of hospitalisations in the past 3 years, number of outpatient visits and prescriptions in the past year and receipt of the prior season’s influenza vaccination. Likelihood ratio tests showed that these confounders were collectively statistically significant (p < 0.05), warranting adjusting for in the models. Further, assessments of model fit using AIC values supported conditioning for PHU region and index week. Detailed results of the model-building can be found in the Supplement. 

Individuals with longer time-since-vaccination intervals were older and more likely to live in rural areas, to be tested for influenza in later months, to have comorbidities and to have received the prior season’s influenza vaccine. Characteristics comparing individuals in different time-since-vaccination groups are tabulated in Supplementary Table S2.

Compared with controls, cases were older and more likely to be living in rural areas and to be previously vaccinated, but less likely to have comorbidities or prior healthcare utilisation. Characteristics of controls and cases, overall and by influenza type/subtype, are appended in Supplementary Table S3. 

**Figure 1:**
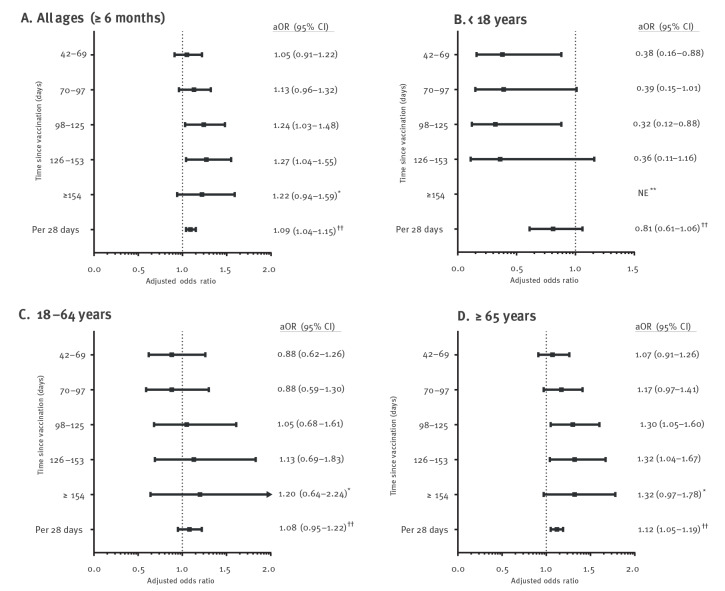
Adjusted odds ratios for any laboratory-confirmed influenza infection by time since seasonal influenza vaccination (categorised into intervals and divided by 28 days) in community-dwelling individuals aged ≥ 6 months, Ontario, Canada, seasons 2010/11 to 2018/19 (n = 53,065)

Using the entire cohort of individuals aged ≥ 6 months, we observed that, compared with the reference interval of 14–41 days, the odds of any laboratory-confirmed influenza infection increased from 1.05 (95% CI: 0.91–1.22) at 42–69 days after vaccination to 1.13 (95% CI: 0.96–1.32) at 70–97 days, 1.24 (95% CI: 1.03–1.48) at 98–125 days, 1.27 (95% CI: 1.04–1.55) at 126–153 days and 1.22 (95% CI: 0.94–1.59) at ≥ 154 days (Figure 1). There was a significant trend associated with each incremental time-since-vaccination interval. In addition, time since vaccination per 28 days after vaccination was linearly associated with the logit of the outcome and we found that the odds of influenza increased by 9% every 28 days (aOR = 1.09; 95% CI: 1.04–1.15). However, there were different patterns across age groups. Notably for those aged < 18 years, we did not observe waning protection and found lower odds of influenza at subsequent intervals. For those aged 18–64 years, point estimates suggested that the odds of influenza increased for longer time-since-vaccination intervals, but all 95% CIs included 1.0. Older adults aged ≥ 65 years had a 32% increase in the odds of influenza at 126–153 days after vaccination (aOR = 1.32; 95% CI: 1.04–1.67) and a 12% increase every 28 days (aOR = 1.12; 95% CI: 1.05–1.19). Counts and unadjusted and adjusted OR estimates for any laboratory-confirmed influenza by time-since-vaccination intervals and per 28 days by age group can be found in Supplementary Table S4.

aOR: adjusted odds ratio; CI: confidence interval; NE: not estimatable.

Cochran–Armitage trend tests were conducted to determine the association between time-since-vaccination group (modelled as a continuous variable) and laboratory-confirmed influenza. The p values for trend tests were marked on the ≥ 154 days estimate. A p value < 0.05 implies a trend between time-since-vaccination categories and laboratory-confirmed influenza.

*p < 0.05.

** p ≥ 0.05.

Estimates with < 6 vaccinated cases (or where the reference group had < 6) were replaced with NE (not estimatable). Time since vaccination per 28 days was modelled using restricted cubic splines and its regression coefficients were used to test for linearity. The p values for linearity tests were marked on the calculated estimate. A p value ≥ 0.05 implies that time since vaccination per 28 days is linearly associated with the logit of the outcome.

†p < 0.05.

††p ≥ 0.05.

**Figure 2:**
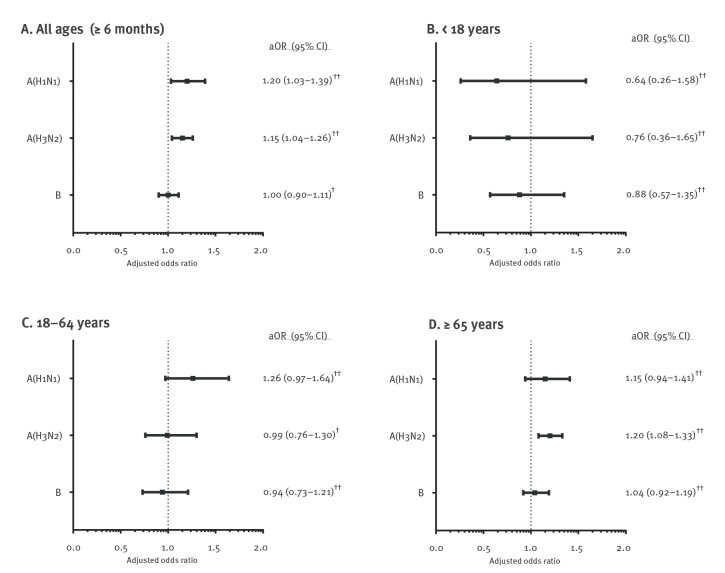
Adjusted odds ratios for every 28 days since seasonal influenza vaccination for any laboratory-confirmed influenza infection and by influenza type/subtype in community-dwelling individuals aged ≥ 6 months, Ontario, Canada, seasons 2010/11 to 2018/19 (n = 48,702)

We also observed variability between age groups against specific influenza types/subtypes; for individuals aged 18–64 years, the point estimate for the odds of influenza per 28 days was highest for influenza A(H1N1) (aOR = 1.26; 95% CI: 0.97–1.64) (Figure 2). For individuals aged ≥ 65 years, the odds of influenza per 28 days was highest for influenza A(H3N2) (aOR = 1.20; 95% CI: 1.08–1.33). The detailed counts and unadjusted and adjusted aOR estimates for each time-since-vaccination group by influenza type/subtype and age group are made available in Supplementary Table S5.

aOR: adjusted odds ratio; CI: confidence interval.

Time since vaccination per 28 days was modelled using restricted cubic splines and its regression coefficients were used to test for linearity. The p values for linearity tests were marked on the calculated estimate. A p value ≥ 0.05 implies that time since vaccination per 28 days is linearly associated with the logit of the outcome.

† p < 0.05.

†† p ≥ 0.05.

**Figure 3:**
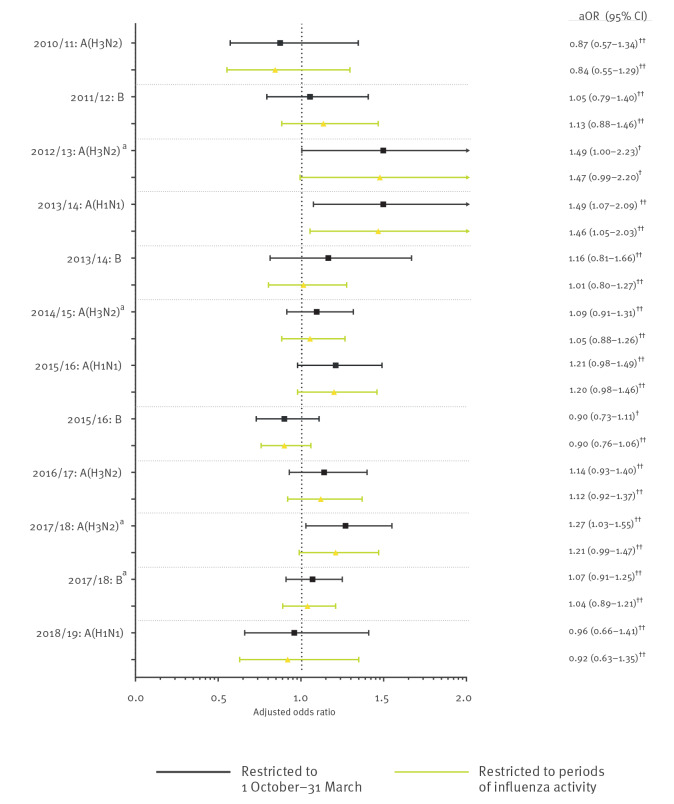
Adjusted odds ratios for every 28 days since seasonal influenza vaccination for the predominant influenza strain(s) in community-dwelling individuals aged ≥ 6 months, Ontario, Canada, seasons 2010/11 to 2018/19 (n = 53,065)

We also observed variability by influenza season (Figure 3). Estimates were insensitive to changes in the definition and length of influenza season (i.e. whether using 1 October to 31 March or season-specific periods of influenza activity). Counts and unadjusted and adjusted OR estimates for the predominant strain(s) for each study season can be found in Supplementary Table S6. Also, the combined seasons’ odds of any laboratory-confirmed influenza infection per 28 days did not change substantially after changing the influenza season definition (aOR = 1.06; 95% CI: 1.01–1.12).

aOR: adjusted odds ratio; CI: confidence interval.

^a^ Influenza seasons with known vaccine mismatch (and consequently low influenza vaccine effectiveness) in Canada.

Time since vaccination per 28 days was modelled using restricted cubic splines and its regression coefficients were used to test for linearity. The p values for linearity tests were marked on the calculated estimate. A p value ≥ 0.05 implies that time since vaccination per 28 days is linearly associated with the logit of the outcome.

† p < 0.05.

†† p ≥ 0.05.

In our negative tracer outcome analyses, we did not observe any associations between time-since-influenza vaccination intervals and laboratory-confirmed RSV infection. The aOR per 28 days for RSV infection was 0.99 (95% CI: 0.90–1.08) (Table). Similarly in our negative tracer exposure analyses among unvaccinated individuals, the 95% CIs included 1.0 (i.e. no association) for all time-since-pseudo-vaccination intervals except for 126–153 days. However, the aOR per 28 days for influenza was significantly less than 1.0 (aOR = 0.95; 95% CI: 0.92–0.98).

**Table:** Odds ratios for negative tracer analyses in community-dwelling individuals aged ≥ 6 months Ontario, Canada, seasons 2010/11 to 2018/19

Time since vaccination (days)	n	n positive	% positive	Unadjusted OR (95% CI)	Adjusted OR (95% CI)
Negative tracer *outcome* analysis between time since influenza vaccination and laboratory-confirmed RSV infection (n = 34,917)					
14–41	5,213	309	6	Reference	
42–69	7,829	747	10	0.88 (0.73–1.06)	0.96 (0.79–1.15)
70–97	8,705	827	10	0.80 (0.65–1.00)	0.91 (0.73–1.14)
98–125	7,160	600	8	0.72 (0.55–0.93)	0.84 (0.65–1.10)
126–153	5,125	371	7	0.73 (0.54–1.00)	0.90 (0.65–1.23)
≥ 154^a^	885	53	6	0.71 (0.45–1.12)*	0.88 (0.55–1.40)*
Per 28 days^b^				0.92 (0.85–1.00)^††^	0.99 (0.90–1.08)^††^
Negative tracer *exposure* analysis between time since pseudo-vaccination date and any laboratory-confirmed influenza infection among unvaccinated individuals (n = 103,939)					
14–41	22,678	1,829	8	Reference	
42–69	21,799	5,768	26	0.96 (0.89–1.04)	1.00 (0.92–1.08)
70–97	25,650	7,863	31	0.91 (0.83–1.00)	0.96 (0.88–1.05)
98–125	19,234	5,583	29	0.85 (0.76–0.94)	0.92 (0.83–1.02)
126–153	13,014	3,100	24	0.75 (0.67–0.85)	0.84 (0.75–0.95)
≥ 154^a^	1,564	360	23	0.75 (0.63–0.89)*	0.84 (0.71–1.00)*
Per 28 days^b^				0.92 (0.89–0.95)^††^	0.95 (0.92–0.98)^††^

CI: confidence interval; OR: odds ratio; RSV: respiratory syncytial virus.

^a^ Cochran-Armitage trend tests were conducted to determine the association between time-since-vaccination/pseudo-vaccination group (a categorical variable modelled as a continuous variable) and laboratory-confirmed influenza/RSV. The p values for linearity tests were marked on the estimate for the last interval (≥ 154 days). A p value < 0.05 implies a trend between time-since-vaccination/pseudo-vaccination categories and laboratory-confirmed influenza/RSV.

*p < 0.05.

** p ≥ 0.05.

^b^ Time since vaccination/pseudo-vaccination per 28 days was modelled using restricted cubic splines and its regression coefficients were used to test for linearity. The p values for trend tests were marked on the calculated estimate. A p value ≥ 0.05 implies that time since vaccination/pseudo-vaccination per 28 days is linearly associated with the logit of the outcome (i.e. accepting the null hypothesis that the restricted cubic spline coefficients are zero).

†p < 0.05.

††p ≥ 0.05.

## Discussion

We found that in community-dwelling individuals aged ≥ 6 months who were vaccinated against influenza during the 2010/11 to 2018/19 seasons in Ontario, Canada, the odds of laboratory-confirmed influenza increased 1.09-times every 28 days since vaccination. The extent of declining vaccine protection varied by influenza type/subtype, age group and influenza season.

Our results were comparable with other vaccinee-only studies examining protection by time since influenza vaccination. A study from Singapore (which has year-round influenza activity due to its tropical climate) found that the infection odds increased 1.07 times every 8 weeks since vaccination [8]. A study in the US found that the odds of influenza A(H3N2) infection increased 1.12 times every 14 days [16]. Another US study found that the odds of influenza increased 1.16 times every 28 days [9].

Intraseasonal declines in vaccine protection could arise from decreasing antibody levels after vaccination, which is impacted by age. Unlike Belongia et al., who found that young children aged 2 years had 1.20 times higher odds of influenza A(H3N2) every 14 days after vaccination [16], we did not observe that children and adolescents aged < 18 years had increased odds of influenza over time. This result is consistent with immunogenicity studies which found that children have persistent antibody responses after vaccination that last throughout an influenza season [17]. However, a recent vaccine effectiveness (VE) study showed that the odds of influenza-related hospitalisation increased 4.2% per month among vaccinated children aged < 18 years [18]. Older adults also had persistent antibody responses [17] but like Belongia et al. [16], we found that older adults had higher odds of influenza over time. These inconsistencies support that post-vaccination antibody levels as a correlate of protection may be different between children and adults [19]. Nonetheless, we cannot conclude that there were true differences in the extent of waning protection between age groups because of overlapping CIs.

We observed that adults aged 18–64 years had a more noticeable decline in vaccine protection against influenza A(H1N1) compared with older adults. Seroconversion after vaccination is inversely proportional to pre-immunisation antibody levels [1,20], thus individuals with previous exposure to particular influenza strains (either through influenza infection or vaccination) might not mount sufficiently high antibody levels *in the current season* to observe declines over time. Younger adults with limited exposure to previous influenza A(H1N1) strains may attain higher antibody levels against A(H1N1) after vaccination compared with older adults who have longer and more complex influenza exposure histories [1,17,21,22] and consequently, waning of protection against influenza A(H1N1) may be more apparent for younger adults than older adults. However, without serology or lineage information in our data, we cannot assess how prior exposure and immunity influence waning vaccine protection.

There was variability in the presence and magnitude of waning protection across study seasons. Trends suggesting waning protection were even observed in seasons with low VE in Canada because of vaccine mismatch (e.g. 2012/13, 2014/15 and 2017/18) [23-25]. However, actual waning might be marginal when the absolute VE is suboptimal [8,16]. Nonetheless, seasonal variation in waning vaccine protection is expected because of differences in the current and prior seasons’ influenza vaccine strain components and circulating strain(s), intraseasonal antigenic drift (which can contribute to waning protection) [26] and the weeks of influenza activity. We cannot conclude whether our seasonal trends differed because of overlapping CIs.

By linking health administrative data to laboratory results for nine influenza seasons, we assembled a large cohort of vaccinated individuals to examine waning influenza vaccine protection in Ontario, Canada. However, our study has several limitations. Firstly, we used specimen collection dates instead of symptom onset dates (which were not available in our data) to calculate time since vaccination, which prolongs the time-since-vaccination interval as testing is later than symptom onset and can increase the likelihood of false negatives. Secondly, specimens used in this study are mostly collected in inpatient settings [10] and the interpretation of the findings might be limited to severe outcomes. Thirdly, we did not have antigenic characterisation data to account for intraseasonal antigenic drift. Fourthly, we were unable to account for vaccine type (TIV/QIV (including LAIV)) since this information is only available for individuals vaccinated in pharmacies (22% of our cohort). This limitation is particularly important for the 2017/18 season, which had a late influenza B Yamagata season while the TIV contained the Victoria lineage component. Fifthly, we excluded individuals vaccinated in settings such as community health centres, public health and workplace clinics because we could not identify them in our vaccination data. However, we do not anticipate that individuals vaccinated in these settings differ with respect to when they received the vaccine, got tested or tested positive compared with those vaccinated in physician offices and pharmacies. Sixthly, our databases do not document prior not laboratory-confirmed infections or have records of all previous seasons’ vaccinations. If repeat vaccinees and those previously infected are more motivated to be vaccinated earlier in the season and had high pre-immunisation antibody levels [1], the association between time since vaccination and influenza could be biased. Lastly, the observational design of our study may be subject to biases. However, we found no association between time since influenza vaccination and RSV infection, implying that residual confounding was minimal. Conversely, there was a decrease in the odds of influenza with increasing time since pseudo-vaccination date in unvaccinated individuals, which was not the association (or direction) we expected. However, this sensitivity analysis probably included individuals who received the influenza vaccine but were misclassified in our data [27]. The collective impact of those with misclassified vaccination status (i.e. decreasing the likelihood of infection) may explain why we observed an aOR < 1.0. Nonetheless the upper CI was close to 1.0.

## Conclusion

We observed that protection from influenza vaccination declines during an influenza season, and that certain age groups may experience more pronounced waning of the protection against specific influenza types/subtypes. Annual influenza vaccination programmes need to strike the balance between vaccinating the population too early versus too late, while taking into account system vaccination capacity and year-to-year variability of influenza season timing. Optimal timing of annual influenza vaccination may improve protection during epidemic waves. 

## Disclaimers

This document used data adapted from the Statistics Canada Postal Code^OM^ Conversion File, which is based on data licensed from Canada Post Corporation, and/or data adapted from the Ontario Ministry of Health Postal Code Conversion File, which contains data copied under license from ^©^Canada Post Corporation and Statistics Canada. Parts of this material are based on data and/or information compiled and provided by the Canadian Institute for Health Information, MOH, and by Ontario Health (OH). However, the analyses, conclusions, opinions and statements expressed herein are solely those of the authors, and do not reflect those of the funding or data sources; no endorsement is intended or should be inferred.

## Supplementary Data

447000 Supplement
