# Supplementary material for: Measuring waning protection from seasonal influenza vaccination during nine influenza seasons, Ontario, Canada, 2010/11 to 2018/19
Source: Euro Surveill. 2024 Feb 22;29(8):2300239. doi: 10.2807/1560-7917.ES.2024.29.8.2300239 (PMC10899815; doi:10.2807/1560-7917.ES.2024.29.8.2300239)
Supplement: Supplement [file 23-00239_KWONG_Supplement.pdf]

Supplementary Material

This supplementary material is hosted by *Eurosurveillance* as supporting information alongside the article '*Measuring waning immunity from seasonal influenza vaccination during nine influenza seasons in Ontario, Canada*', on behalf of the authors, who remain responsible for the accuracy and appropriateness of the content. The same standards for ethics, copyright, attributions and permissions as for the article apply. Supplements are not edited by *Eurosurveillance* and the journal is not responsible for the maintenance of any links or email addresses provided therein.

Supplementary Methods 2

Supplementary Table S1. Characteristics of the 2010-2011 to 2018-2019 influenza seasons in Ontario, Canada. 2

Supplementary Results 4

Results of model-building exercises. 4

Supplementary Table S2. Characteristics of community-dwelling individuals aged ≥6 months vaccinated against seasonal influenza before influenza testing by time since vaccination during the 2010-2011 to 2018-2019 influenza seasons in Ontario, Canada. 6

Supplementary Table S3. Characteristics of community-dwelling individuals aged ≥6 months vaccinated against seasonal influenza before influenza testing during the 2010-2011 to 2018-2019 influenza seasons in Ontario, Canada, by influenza status and by type/subtype[a]. 9

Supplementary Table S4. Odds ratios (OR) by time since vaccination (TSV) against any laboratory-confirmed influenza infection in community-dwelling individuals aged ≥6 months in Ontario, Canada during the 2010-2011 to 2018-2019 season, by age groups. 12

Supplementary Table S5. Odds ratios (OR) by time since vaccination (TSV) against laboratory-confirmed influenza by type/subtype in community-dwelling individuals aged ≥6 months in Ontario, Canada during the 2010-2011 to 2018-2019 season, by age groups. 13

Supplementary Table S6. Odds ratios (OR) by time since vaccination (TSV) against laboratory-confirmed influenza infection of the predominant circulating strain(s) in community-dwelling individuals aged ≥6 months in Ontario, Canada by influenza season. 15

Supplementary Methods

Supplementary Table S1. Characteristics of the 2010-2011 to 2018-2019 influenza seasons in Ontario, Canada.

	2010-2011	2011-2012	2012-2013[a]	2013-2014	2014-2015[a]	2015-2016	2016-2017	2017-2018[a]	2018-2019
**Influenza season start and end dates[b]**									
Start	November 14, 2010 (Week 46)	January 29, 2012 (Week 5)	November 4, 2012 (Week 45)	December 1, 2013 (Week 49)	November 23, 2014 (Week 48)	January 17, 2016 (Week 3)	November 20, 2016 (Week 47)	November 26, 2017 (Week 48)	November 25, 2018 (Week 48)
End	April 9, 2011 (Week 14)	May 19, 2012 (Week 20)	May 11, 2013 (Week 19)	May 24, 2014 (Week 21)	May 16, 2015 (Week 19)	May 28, 2016 (Week 21)	May 27, 2017 (Week 21)	May 12, 2018 (Week 19)	May 25, 2019 (Week 21)
**Strains in vaccines licensed for use in Ontario[c]**									
H1N1	A/California/7/2009 (NYMC X-179A)	A/California/7/2009 (NYMC X-179A or X-181)	A/California/7/2009 (NYMC X-179A)	A/California/7/2009 (NYMC X-179A)	A/California/7/2009 (NYMC X-179A)	A/California/7/2009 (NYMC X-179A)	A/California/7/2009 (NYMC X-179A or X-181)	A/Singapore/GP1908/2015 (IVR-180) *(A/Michigan/45/2015 (H1N1)pdm09-like)*	A/Singapore/GP1908/2015 (IVR-180) *(A/Michigan/45/2015 (H1N1)pdm09-like)*
H3N2	A/Victoria/210/2009 (NYMC X-187)	A/Victoria/210/2009 (NYMC X-187)	A/Victoria/361/2011 (IVR-165)	A/Texas/50/2012 (NYMC X-223A)	A/Texas/50/2012 (NYMC X-223A)	A/Switzerland/9715293/201 (NIB-88)	A/Hong Kong/4801/2014 (NYMC X-263B)	A/Hong Kong/4801/2014 (NYMC X-263B)	A/Singapore/INFIMH-16-0019/2016 (IVR-186 or NIB-104)
B (Victoria)	B/Brisbane/60/2008	B/Brisbane/60/2008				B/Brisbane/60/2008	B/Brisbane/60/2008	B/Brisbane/60/2008	B/Maryland/15/2016 (NYMC BX-69A) *(B/Colorado/06/2017-like virus)*
B (Yamagata)			B/Hubei-Wujiagang/158/2009 (NYMC BX-39)	B/Massachusetts/2/2012 (NYMC BX-51B)	B/Massachusetts/2/2012 (NYMC BX-51B)	B/Phuket/3073/2013	B/Phuket/3073/2013	B/Phuket/3073/2013	B/Phuket/3073/2013
**Influenza type/subtype distribution reported for national surveillance by Ontario[d]**									
H1N1	9%	16%	8%	54%	1%	65%	1%	6%	60%
H3N2	80%	10%	82%	10%	87%	8%	87%	51%	35%
B (Victoria)	10%	37%	2%	1%	1%	21%	2%	2%	4%
B (Yamagata)	1%	37%	8%	35%	11%	6%	9%	41%	1%
**Influenza type/subtype distribution in Ontario's health administrative databases (all ages)[e]**									
H1N1	7%	16%	8%	51%	<1%	60%	<1%	4%	50%
H3N2	80%	9%	81%	9%	86%	8%	86%	51%	46%
B	13%	75%	11%	41%	14%	32%	13%	45%	4%

[a] Influenza seasons with known vaccine mismatch (and consequently low influenza vaccine effectiveness) in Canada.

[b] The start of the influenza season was determined to be the beginning of the week (Sunday) when the proportion of individuals testing positive for influenza exceeded 5%. The end of the season was Saturday prior to the week when the proportion of influenza-positive individuals fell below 5%.
[c] Information on the strains in the influenza vaccines available in Ontario is from the product monographs of the vaccines from each respective influenza season.
[d] Proportions were calculated based on the end-of-season total number of positive influenza specimens by type and subtype in Ontario according to the weekly influenza reports from the Public Health Agency of Canada. Unsubtyped influenza A cases were assumed to have the same distribution as subtyped influenza A cases and were re-classified accordingly. Similarly, influenza B cases were assumed to have the same lineage distribution as influenza B cases in Canada that were antigenetically characterized by the National Microbiology Laboratory and were re-classified accordingly.
[e] Approximately 49% of all influenza A specimens in Ontario's health administrative databases were subtyped. Unsubtyped influenza A cases were assumed to have the same distribution as subtyped influenza A cases and were re-classified accordingly. Lineage information was not available for majority of influenza B cases.

Supplementary Results

Results of model-building exercises.

A. Identifying confounders in the association between time-since-vaccination groups and laboratory-confirmed influenza:

Characteristic	P-value of Type 3 Analysis: Univariate association between characteristic* and time-since-vaccination group (multinomial logistic regression)	P-value of Type 3 Analysis: Univariate association between characteristic* and laboratory-confirmed influenza (logistic regression)
**Age group**	**<.0001**	**<.0001**
Sex	0.5433	0.0014
Neighbourhood income quintile	0.0560	0.0332
Rurality	0.0281	0.0984
**Number of hospitalizations in the past 3 years**	**<.0001**	**<.0001**
**Number of physician office visits in the past year**	**<.0001**	**<.0001**
**Number of prescriptions in the past year**	**0.0001**	**<.0001**
Receipt of home care in the past year	0.6543	<.0001
**Any comorbidity**	**0.0006**	**<.0001**
**Receipt of prior season's vaccination**	**<.0001**	**<.0001**

*Variables bolded were statistically significant ($p<0.05$) in univariate associations with the exposure (time-since-vaccination group) and outcome (laboratory-confirmed influenza) and were included in the main model as confounders.

B. Assessing the percent change in the beta-coefficients for time-since-vaccination groups when adjusting for each confounder in a logistic regression model:

	Unadjusted	Age group		Number of hospitalizations in the past 3 years		Number of prescriptions in the past year		Number of physician office visits in the past year		Any comorbidity		Receipt of prior season's vaccination	
	β	β	% Change	β	% Change	β	% Change	β	% Change	β	% Change	β	% Change
42-69 days	1.23	1.22	0.84	1.22	0.86	1.23	0.25	1.23	0.16	1.23	0.36	1.23	0.14
70-97 days	1.42	1.41	0.74	1.41	0.82	1.43	0.44	1.42	0.26	1.42	0.34	1.42	0.16
98-125 days	1.37	1.36	0.38	1.35	1.40	1.37	0.15	1.37	0.14	1.37	0.33	1.37	0.16
126-153 days	1.20	1.19	0.90	1.18	1.60	1.21	0.58	1.20	0.49	1.21	0.74	1.20	0.27
≥154 days	1.03	1.02	1.05	1.01	1.81	1.03	0.62	1.05	1.96	1.04	0.96	1.02	0.39

C. Likelihood ratio test (LRT) comparing unadjusted model and model adjusting for covariates:

Model	-2 Log L	DF	P-value of LRT
Unadjusted	51077.319	5	<0.0001
Adjusted for confounders	50599.589	15	

D. Comparing Akaike's Information Criteria (AIC) values:

Model	AIC
Adjusted for confounders only	50631.589
Adjusted for confounders and conditioning for Public Health Unit region and week of influenza test	41421.763

Supplementary Table S2. Characteristics of community-dwelling individuals aged ≥6 months vaccinated against seasonal influenza before influenza testing by time since vaccination during the 2010-2011 to 2018-2019 influenza seasons in Ontario, Canada.

	Time since vaccination to specimen collection date					
**Characteristic**	**14-41 days N=6,774**	**42-69 days N=11,987**	**70-97 days N=13,947**	**98-125 days N=11,255**	**126-153 days N=7,782**	**≥154 days N=1,320**
Time since vaccination to specimen collection date (days), mean +/- standard deviation	28.56 ± 8.09	56.69 ± 7.97	83.05 ± 8.07	111.18 ± 8.12	137.72 ± 7.78	160.36 ± 5.30
Positive for influenza by real-time polymerase chain reaction	457 (6.7%)	2,377 (19.8%)	3,210 (23.0%)	2,493 (22.2%)	1,505 (19.3%)	222 (16.8%)
Influenza A/H1N1[a]	36 (7.9%)	136 (5.7%)	196 (3.1%)	168 (3.4%)	110 (7.3%)	17 (7.7%)
Influenza A/H3N2[a]	219 (47.9%)	1,040 (43.8%)	1,139 (18.0%)	680 (13.6%)	334 (22.2%)	36 (16.2%)
Influenza B[a]	44 (9.6%)	215 (9.0%)	415 (6.6%)	566 (11.4%)	465 (30.9%)	85 (38.3%)
Missing influenza type/subtype information[a]	158 (34.6%)	986 (41.5%)	4,568 (72.3%)	3,571 (71.6%)	596 (39.6%)	84 (37.8%)
Age (years), mean +/- standard deviation	68.83 ± 21.99	72.14 ± 19.19	72.36 ± 18.82	71.81 ± 18.73	73.02 ± 17.67	73.34 ± 16.37
Age group						
6 months-17 years	425 (6.3%)	430 (3.6%)	472 (3.4%)	386 (3.4%)	206 (2.6%)	27 (2.0%)
18-49 years	478 (7.1%)	688 (5.7%)	792 (5.7%)	648 (5.8%)	409 (5.3%)	64 (4.8%)
50-64 years	992 (14.6%)	1,615 (13.5%)	1,867 (13.4%)	1,612 (14.3%)	1,041 (13.4%)	194 (14.7%)
65-74 years	1,513 (22.3%)	2,601 (21.7%)	3,008 (21.6%)	2,566 (22.8%)	1,751 (22.5%)	283 (21.4%)
75-84 years	1,864 (27.5%)	3,525 (29.4%)	4,184 (30.0%)	3,313 (29.4%)	2,371 (30.5%)	415 (31.4%)
≥85 years	1,502 (22.2%)	3,128 (26.1%)	3,624 (26.0%)	2,730 (24.3%)	2,004 (25.8%)	337 (25.5%)
Sex						
Female	3,440 (50.8%)	6,122 (51.1%)	7,237 (51.9%)	5,715 (50.8%)	3,990 (51.3%)	673 (51.0%)
Male	3,334 (49.2%)	5,865 (48.9%)	6,710 (48.1%)	5,540 (49.2%)	3,792 (48.7%)	647 (49.0%)
Rural residence	382 (5.6%)	758 (6.3%)	958 (6.9%)	764 (6.8%)	542 (7.0%)	103 (7.8%)
Public Health Unit region						
Central East	411 (6.1%)	696 (5.8%)	897 (6.4%)	753 (6.7%)	515 (6.6%)	95 (7.2%)
Central West	1,330 (19.6%)	2,106 (17.6%)	2,608 (18.7%)	2,130 (18.9%)	1,457 (18.7%)	224 (17.0%)
Durham	234 (3.5%)	425 (3.5%)	423 (3.0%)	353 (3.1%)	269 (3.5%)	34 (2.6%)
Eastern	223 (3.3%)	505 (4.2%)	597 (4.3%)	434 (3.9%)	309 (4.0%)	62 (4.7%)
North	228 (3.4%)	483 (4.0%)	598 (4.3%)	489 (4.3%)	347 (4.5%)	49 (3.7%)
Ottawa	367 (5.4%)	704 (5.9%)	818 (5.9%)	732 (6.5%)	496 (6.4%)	68 (5.2%)
Peel	719 (10.6%)	1,269 (10.6%)	1,442 (10.3%)	1,214 (10.8%)	754 (9.7%)	132 (10.0%)

South West	808 (11.9%)	1,469 (12.3%)	1,702 (12.2%)	1,478 (13.1%)	1,078 (13.9%)	211 (16.0%)
Toronto	2,004 (29.6%)	3,518 (29.3%)	3,917 (28.1%)	2,916 (25.9%)	2,067 (26.6%)	351 (26.6%)
York	437 (6.5%)	786 (6.6%)	914 (6.6%)	732 (6.5%)	469 (6.0%)	90 (6.8%)
Missing information	13 (0.2%)	26 (0.2%)	31 (0.2%)	24 (0.2%)	21 (0.3%)	≤5 (0.4%)
Neighbourhood income quintile						
1 (lowest)	1,686 (24.9%)	2,953 (24.6%)	3,379 (24.2%)	2,772 (24.6%)	1,911 (24.6%)	321 (24.3%)
2	1,416 (20.9%)	2,685 (22.4%)	3,170 (22.7%)	2,433 (21.6%)	1,730 (22.2%)	300 (22.7%)
3	1,311 (19.4%)	2,293 (19.1%)	2,686 (19.3%)	2,136 (19.0%)	1,440 (18.5%)	231 (17.5%)
4	1,113 (16.4%)	1,911 (15.9%)	2,336 (16.7%)	1,935 (17.2%)	1,250 (16.1%)	201 (15.2%)
5 (highest)	1,228 (18.1%)	2,106 (17.6%)	2,329 (16.7%)	1,942 (17.3%)	1,424 (18.3%)	260 (19.7%)
Missing information	20 (0.3%)	39 (0.3%)	47 (0.3%)	37 (0.3%)	27 (0.3%)	7 (0.5%)
Season of specimen collection						
2010-2011	211 (3.1%)	485 (4.0%)	671 (4.8%)	460 (4.1%)	342 (4.4%)	47 (3.6%)
2011-2012	386 (5.7%)	513 (4.3%)	505 (3.6%)	519 (4.6%)	412 (5.3%)	131 (9.9%)
2012-2013	239 (3.5%)	665 (5.5%)	905 (6.5%)	523 (4.6%)	386 (5.0%)	109 (8.3%)
2013-2014	596 (8.8%)	1,057 (8.8%)	1,273 (9.1%)	915 (8.1%)	756 (9.7%)	176 (13.3%)
2014-2015	936 (13.8%)	2,014 (16.8%)	2,271 (16.3%)	1,459 (13.0%)	931 (12.0%)	178 (13.5%)
2015-2016	982 (14.5%)	1,226 (10.2%)	1,415 (10.1%)	1,790 (15.9%)	1,321 (17.0%)	161 (12.2%)
2016-2017	881 (13.0%)	2,008 (16.8%)	2,099 (15.0%)	1,479 (13.1%)	983 (12.6%)	108 (8.2%)
2017-2018	1,227 (18.1%)	2,166 (18.1%)	2,800 (20.1%)	2,481 (22.0%)	1,479 (19.0%)	215 (16.3%)
2018-2019	1,316 (19.4%)	1,853 (15.5%)	2,008 (14.4%)	1,629 (14.5%)	1,172 (15.1%)	195 (14.8%)
Month of specimen collection						
October	86 (1.3%)	0 (0.0%)	0 (0.0%)	0 (0.0%)	0 (0.0%)	0 (0.0%)
November	3,042 (44.9%)	248 (2.1%)	0 (0.0%)	0 (0.0%)	0 (0.0%)	0 (0.0%)
December	3,344 (49.4%)	6,462 (53.9%)	1,050 (7.5%)	0 (0.0%)	0 (0.0%)	0 (0.0%)
January	261 (3.9%)	5,050 (42.1%)	9,522 (68.3%)	1,346 (12.0%)	0 (0.0%)	0 (0.0%)
February	41 (0.6%)	190 (1.6%)	3,172 (22.7%)	6,933 (61.6%)	1,061 (13.6%)	0 (0.0%)
March	0 (0.0%)	37 (0.3%)	203 (1.5%)	2,976 (26.4%)	6,721 (86.4%)	1,320 (100.0%)
Medical comorbidities						
Anemia	1,441 (21.3%)	2,560 (21.4%)	2,978 (21.4%)	2,393 (21.3%)	1,711 (22.0%)	308 (23.3%)
Asthma	2,233 (33.0%)	3,813 (31.8%)	4,527 (32.5%)	3,585 (31.9%)	2,476 (31.8%)	422 (32.0%)
Cancer	1,858 (27.4%)	3,369 (28.1%)	3,884 (27.8%)	3,217 (28.6%)	2,305 (29.6%)	399 (30.2%)
Ischemic heart disease	2,164 (31.9%)	4,062 (33.9%)	4,749 (34.1%)	3,731 (33.1%)	2,653 (34.1%)	436 (33.0%)
Arrhythmia	1,739 (25.7%)	3,388 (28.3%)	3,848 (27.6%)	3,048 (27.1%)	2,100 (27.0%)	361 (27.3%)
Congestive heart failure	2,400 (35.4%)	4,379 (36.5%)	5,078 (36.4%)	3,964 (35.2%)	2,818 (36.2%)	457 (34.6%)
Chronic kidney disease	1,433 (21.2%)	2,562 (21.4%)	2,956 (21.2%)	2,382 (21.2%)	1,745 (22.4%)	291 (22.0%)

Chronic obstructive pulmonary disease	3,227 (47.6%)	5,732 (47.8%)	6,717 (48.2%)	5,315 (47.2%)	3,829 (49.2%)	686 (52.0%)
Diabetes	2,615 (38.6%)	4,914 (41.0%)	5,796 (41.6%)	4,644 (41.3%)	3,219 (41.4%)	547 (41.4%)
Dementia/frailty	991 (14.6%)	1,861 (15.5%)	2,185 (15.7%)	1,687 (15.0%)	1,175 (15.1%)	193 (14.6%)
Immunocompromise	1,112 (16.4%)	1,849 (15.4%)	2,053 (14.7%)	1,682 (14.9%)	1,225 (15.7%)	201 (15.2%)
History of transient ischemic attack or stroke	662 (9.8%)	1,336 (11.1%)	1,541 (11.0%)	1,134 (10.1%)	838 (10.8%)	135 (10.2%)
Any childhood complex chronic condition[b]	94 (36.3%)	95 (35.7%)	86 (34.3%)	65 (32.8%)	44 (44.9%)	8 (80.0%)
Any of the above comorbidities	6,263 (92.5%)	11,163 (93.1%)	12,990 (93.1%)	10,476 (93.1%)	7,330 (94.2%)	1,247 (94.5%)
Number of hospitalizations in the past 3 years, mean +/- standard deviation	1.90 ± 2.81	1.69 ± 2.61	1.65 ± 2.41	1.56 ± 2.36	1.56 ± 2.20	1.57 ± 2.14
Number of physician office visits in the past year, mean +/- standard deviation	15.39 ± 11.15	15.40 ± 11.35	15.53 ± 11.69	15.37 ± 11.32	15.75 ± 11.53	17.22 ± 14.68
Number of prescriptions in the past year, mean +/- standard deviation	14.26 ± 10.65	14.42 ± 10.18	14.73 ± 10.21	14.29 ± 10.12	14.83 ± 10.09	14.80 ± 10.18
Receipt of home care in the past year	3,021 (44.6%)	5,305 (44.3%)	6,228 (44.7%)	4,950 (44.0%)	3,516 (45.2%)	580 (43.9%)
Receipt of prior season's vaccination	4,791 (70.7%)	8,720 (72.7%)	10,231 (73.4%)	8,246 (73.3%)	5,778 (74.2%)	991 (75.1%)

[a] The proportions by influenza type/subtype (including those missing information) were calculated among individuals positive for influenza for each time-since-vaccination group.

[b] The proportions with 'any children complex chronic condition' was calculated among individuals aged 6-59 months only (n=1,082).

Supplementary Table S3. Characteristics of community-dwelling individuals aged ≥6 months vaccinated against seasonal influenza before influenza testing during the 2010-2011 to 2018-2019 influenza seasons in Ontario, Canada, by influenza status and by type/subtype[a].

Characteristic	Influenza negative N=42,801	Influenza positive N=10,264	Influenza A/H1N1[c] N=663	Influenza A/H3N2 N=3,448	Influenza B N=1,790
Time since vaccination to specimen collection date (days), mean +/- standard deviation	84.77 ± 37.66	91.38 ± 31.71	92.99 ± 32.79	83.31 ± 30.17	106.14 ± 31.69
Time since vaccination group					
14-41 days	6,317 (14.8%)	457 (4.5%)	36 (5.4%)	219 (6.4%)	44 (2.5%)
42-69 days	9,610 (22.5%)	2,377 (23.2%)	136 (20.5%)	1,040 (30.2%)	215 (12.0%)
70-97 days	10,737 (25.1%)	3,210 (31.3%)	196 (29.6%)	1,139 (33.0%)	415 (23.2%)
98-125 days	8,762 (20.5%)	2,493 (24.3%)	168 (25.3%)	680 (19.7%)	566 (31.6%)
126-153 days	6,277 (14.7%)	1,505 (14.7%)	110 (16.6%)	334 (9.7%)	465 (26.0%)
≥154 days	1,098 (2.6%)	222 (2.2%)	17 (2.6%)	36 (1.0%)	85 (4.7%)
Age (years), mean +/- standard deviation	71.54 ± 19.28	73.22 ± 18.46	64.30 ± 19.23	75.21 ± 16.91	71.02 ± 20.59
Age group					
6 months-17 years	1,630 (3.8%)	316 (3.1%)	25 (3.8%)	70 (2.0%)	90 (5.0%)
18-49 years	2,482 (5.8%)	597 (5.8%)	87 (13.1%)	187 (5.4%)	112 (6.3%)
50-64 years	6,090 (14.2%)	1,231 (12.0%)	159 (24.0%)	321 (9.3%)	246 (13.7%)
65-74 years	9,638 (22.5%)	2,084 (20.3%)	155 (23.4%)	666 (19.3%)	365 (20.4%)
75-84 years	12,525 (29.3%)	3,147 (30.7%)	173 (26.1%)	1,128 (32.7%)	503 (28.1%)
≥85 years	10,436 (24.4%)	2,889 (28.1%)	64 (9.7%)	1,076 (31.2%)	474 (26.5%)
Sex					
Female	21,775 (50.9%)	5,402 (52.6%)	348 (52.5%)	1,843 (53.5%)	966 (54.0%)
Male	21,026 (49.1%)	4,862 (47.4%)	315 (47.5%)	1,605 (46.5%)	824 (46.0%)
Rural residence	2,781 (6.5%)	726 (7.1%)	49 (7.4%)	306 (8.9%)	120 (6.7%)
Public Health Unit region					
Central East	2,748 (6.4%)	619 (6.0%)	32 (4.8%)	243 (7.0%)	109 (6.1%)
Central West	8,087 (18.9%)	1,768 (17.2%)	71 (10.7%)	333 (9.7%)	308 (17.2%)
Durham	1,377 (3.2%)	361 (3.5%)	25 (3.8%)	79 (2.3%)	63 (3.5%)
Eastern	1,673 (3.9%)	457 (4.5%)	34 (5.1%)	174 (5.0%)	55 (3.1%)
North	1,715 (4.0%)	479 (4.7%)	35 (5.3%)	243 (7.0%)	60 (3.4%)
Ottawa	2,515 (5.9%)	670 (6.5%)	6 (0.9%)	19 (0.6%)	135 (7.5%)

Peel	4,555 (10.6%)	975 (9.5%)	74 (11.2%)	308 (8.9%)	207 (11.6%)
South West	5,371 (12.5%)	1,375 (13.4%)	78 (11.8%)	315 (9.1%)	308 (17.2%)
Toronto	11,911 (27.8%)	2,862 (27.9%)	259 (39.1%)	1,382 (40.1%)	413 (23.1%)
York	2,755 (6.4%)	673 (6.6%)	47 (7.1%)	343 (9.9%)	128 (7.2%)
Missing information	94 (0.2%)	25 (0.2%)	≤5 (≤0.8%)	9 (0.3%)	≤5 (≤0.3%)
Neighbourhood income quintile					
1 (lowest)	10,413 (24.3%)	2,609 (25.4%)	166 (25.0%)	903 (26.2%)	420 (23.5%)
2	9,456 (22.1%)	2,278 (22.2%)	145 (21.9%)	786 (22.8%)	402 (22.5%)
3	8,154 (19.1%)	1,943 (18.9%)	119 (17.9%)	627 (18.2%)	342 (19.1%)
4	7,036 (16.4%)	1,710 (16.7%)	119 (17.9%)	547 (15.9%)	313 (17.5%)
5 (highest)	7,598 (17.8%)	1,691 (16.5%)	112 (16.9%)	575 (16.7%)	308 (17.2%)
Missing information	144 (0.3%)	33 (0.3%)	≤5 (≤0.8%)	10 (0.3%)	≤5 (≤0.3%)
Season of specimen collection					
2010-2011	1,821 (4.3%)	395 (3.8%)	6 (0.9%)	259 (7.5%)	23 (1.3%)
2011-2012	2,292 (5.4%)	174 (1.7%)	26 (3.9%)	22 (0.6%)	115 (6.4%)
2012-2013	2,249 (5.3%)	578 (5.6%)	26 (3.9%)	313 (9.1%)	19 (1.1%)
2013-2014	4,195 (9.8%)	578 (5.6%)	173 (26.1%)	39 (1.1%)	169 (9.4%)
2014-2015	5,696 (13.3%)	2,093 (20.4%)	0 (0.0%)	856 (24.8%)	93 (5.2%)
2015-2016	5,887 (13.8%)	1,008 (9.8%)	262 (39.5%)	35 (1.0%)	211 (11.8%)
2016-2017	5,898 (13.8%)	1,660 (16.2%)	≤5 (≤0.8%)	1,043 (30.2%)	64 (3.6%)
2017-2018	7,722 (18.0%)	2,646 (25.8%)	13-17 (2.0-2.6%)	685 (19.9%)	1,082 (60.4%)
2018-2019	7,041 (16.5%)	1,132 (11.0%)	152 (22.9%)	196 (5.7%)	14 (0.8%)
Month of specimen collection					
October/November	3,276 (7.7%)	100 (1.0%)	≤5 (≤0.8%)	55 (1.6%)	10 (0.6%)
December	9,009 (21.0%)	1,847 (18.0%)	109-113 (16.4-17.0%)	922 (26.7%)	115 (6.4%)
January	12,555 (29.3%)	3,624 (35.3%)	190 (28.7%)	1,356 (39.3%)	431 (24.1%)
February	8,919 (20.8%)	2,478 (24.1%)	183 (27.6%)	693 (20.1%)	504 (28.2%)
March	9,042 (21.1%)	2,215 (21.6%)	176 (26.5%)	422 (12.2%)	730 (40.8%)
Medical comorbidities					
Anemia	9,391 (21.9%)	2,000 (19.5%)	120 (18.1%)	651 (18.9%)	375 (20.9%)
Asthma	13,814 (32.3%)	3,242 (31.6%)	238 (35.9%)	1,032 (29.9%)	571 (31.9%)
Cancer	12,493 (29.2%)	2,539 (24.7%)	138 (20.8%)	833 (24.2%)	443 (24.7%)
Ischemic heart disease	14,478 (33.8%)	3,317 (32.3%)	191 (28.8%)	1,103 (32.0%)	554 (30.9%)
Arrhythmia	11,742 (27.4%)	2,742 (26.7%)	150 (22.6%)	874 (25.3%)	464 (25.9%)
Congestive heart failure	15,933 (37.2%)	3,163 (30.8%)	175 (26.4%)	1,050 (30.5%)	501 (28.0%)
Chronic kidney disease	9,398 (22.0%)	1,971 (19.2%)	105 (15.8%)	621 (18.0%)	363 (20.3%)

Chronic obstructive pulmonary disease	20,944 (48.9%)	4,562 (44.4%)	302 (45.6%)	1,460 (42.3%)	739 (41.3%)
Diabetes	17,391 (40.6%)	4,344 (42.3%)	273 (41.2%)	1,455 (42.2%)	726 (40.6%)
Dementia/frailty	6,318 (14.8%)	1,774 (17.3%)	68 (10.3%)	636 (18.4%)	284 (15.9%)
Immunocompromise	6,741 (15.7%)	1,381 (13.5%)	98 (14.8%)	395 (11.5%)	273 (15.3%)
History of transient ischemic attack or stroke	4,495 (10.5%)	1,151 (11.2%)	51 (7.7%)	411 (11.9%)	162 (9.1%)
Any childhood complex chronic condition	353 (36.7%)	39 (32.2%)	≤5 (≤33.3%)	≤5 (≤29.4%)	13 (41.9%)
Any of the above comorbidities	40,109 (93.7%)	9,360 (91.2%)	576 (86.9%)	3,093 (89.7%)	1,614 (90.2%)
Number of hospitalizations in the past 3 years, mean +/- standard deviation	1.74 ± 2.55	1.30 ± 2.04	1.26 ± 1.92	1.17 ± 1.71	1.29 ± 2.26
Number of physician office visits in the past year, mean +/- standard deviation	15.80 ± 11.59	14.36 ± 11.19	14.97 ± 12.66	14.45 ± 11.75	14.16 ± 10.93
Number of prescriptions in the past year, mean +/- standard deviation	14.70 ± 10.34	13.78 ± 9.71	12.59 ± 10.87	13.83 ± 9.61	13.08 ± 9.94
Receipt of home care in the past year	19,379 (45.3%)	4,221 (41.1%)	189 (28.5%)	1,409 (40.9%)	702 (39.2%)
Receipt of prior season's vaccination	31,084 (72.6%)	7,673 (74.8%)	431 (65.0%)	2,618 (75.9%)	1,308 (73.1%)

[a] Among the vaccinated cases, 5,901 (57%) had type/subtype information.
[b] The proportions with 'any children complex chronic condition' was calculated among individuals aged 6-59 months only (n=1,082).
[c] Ranges are presented to avoid back calculation of counts ≤5.

Supplementary Table S4. Odds ratios (OR) by time since vaccination (TSV) against any laboratory-confirmed influenza infection in community-dwelling individuals aged ≥6 months in Ontario, Canada during the 2010-2011 to 2018-2019 season, by age groups.

TSV (days)	No. vaccinated	No. influenza-positive (%)[a]	Unadjusted OR (95% CI)	Adjusted OR (95% CI)
**All ages (≥6 months)**				
14-41	6774	457 (7)	Ref.	Ref.
42-69	11987	2377 (20)	1.04 (0.90, 1.20)	1.05 (0.91, 1.22)
70-97	13947	3210 (23)	1.09 (0.93, 1.28)	1.13 (0.96, 1.32)
98-125	11255	2493 (22)	1.19 (1.00, 1.42)	1.24 (1.03, 1.48)
126-153	7782	1505 (19)	1.20 (0.98, 1.47)	1.27 (1.04, 1.55)
≥154[b]	1320	222 (17)	1.15 (0.88, 1.48)**	1.22 (0.94, 1.59)*
Per 28 days[c]			1.07 (1.02, 1.13)††	1.09 (1.04, 1.15)††
**<18 years**				
14-41	425	23 (5)	Ref.	Ref.
42-69	430	57 (13)	0.36 (0.16, 0.84)	0.38 (0.16, 0.88)
70-97	472	105 (22)	0.36 (0.14, 0.91)	0.39 (0.15, 1.01)
98-125	386	80 (21)	0.29 (0.11, 0.77)	0.32 (0.12, 0.88)
126-153	206	46-50 (22-24)	0.32 (0.10, 0.99)	0.36 (0.11, 1.16)
≥154[b]	27	≤5	NE**	NE**
Per 28 days[c]			0.77 (0.59, 1.00)††	0.81 (0.61, 1.06)††
**18-64 years**				
14-41	1470	85 (6)	Ref.	Ref.
42-69	2303	390 (17)	0.81 (0.57, 1.16)	0.88 (0.62, 1.26)
70-97	2659	544 (20)	0.79 (0.54, 1.17)	0.88 (0.59, 1.30)
98-125	2260	491 (22)	0.91 (0.60, 1.40)	1.05 (0.68, 1.61)
126-153	1450	272-276 (19)	0.95 (0.59, 1.53)	1.13 (0.69, 1.83)
≥154[b]	258	44-48 (17-19)	0.98 (0.53, 1.82)**	1.20 (0.64, 2.24)*
Per 28 days[c]			1.04 (0.92, 1.17)††	1.08 (0.95, 1.22)††
**≥65 years**				
14-41	4879	349 (7)	Ref.	Ref.
42-69	9254	1930 (21)	1.06 (0.90, 1.25)	1.07 (0.91, 1.26)
70-97	10816	2561 (24)	1.13 (0.94, 1.36)	1.17 (0.97, 1.41)
98-125	8609	1922 (22)	1.25 (1.01, 1.54)	1.30 (1.05, 1.60)
126-153	6126	1185 (19)	1.25 (0.99, 1.57)	1.32 (1.04, 1.67)
≥154[b]	1035	173 (17)	1.24 (0.92, 1.68)*	1.32 (0.97, 1.78)*
Per 28 days[c]			1.10 (1.03, 1.17)††	1.12 (1.05, 1.19)††

[a] Counts with ≤5 vaccinated cases were suppressed and estimates were replaced with NE (not estimable). Estimates where the reference group (i.e., 14-41 days) had ≤5 vaccinated cases were replaced with NE. Ranges are presented to avoid back calculation of counts ≤5.

[b] Cochran-Armitage trend tests were conducted to determine the association between time since vaccination group (a categorical variable modelled as a continuous variable) and laboratory-confirmed influenza. P-values for trend tests were marked on the estimate for the last interval (≥154 days); *p <0.05; ** p≥0.05. A P-value <0.05 implies a trend between time since vaccination categories and laboratory-confirmed influenza.

[c] Time since vaccination per 28 days were modelled using restricted cubic splines and its regression coefficients were used to test for linearity. P-values for linearity tests were marked on the calculated estimate; †p <0.05; ††p≥0.05. A P-value ≥0.05 implies that time since vaccination per 28 days is linearly associated with the logit of the outcome (i.e., accepting the null hypothesis that the restricted cubic spline coefficients are zero).

Supplementary Table S5. Odds ratios (OR) by time since vaccination (TSV) against laboratory-confirmed influenza by type/subtype in community-dwelling individuals aged ≥6 months in Ontario, Canada during the 2010-2011 to 2018-2019 season, by age groups.

	Influenza A/H1N1				Influenza A/H3N2				Influenza B			
**TSV (days)**	**No. vaccinated**	**No. influenza-positive (%)[a]**	**Unadjusted OR (95% CI)**	**Adjusted OR (95% CI)**	**No. vaccinated**	**No. influenza-positive (%)[a]**	**Unadjusted OR (95% CI)**	**Adjusted OR (95% CI)**	**No. vaccinated**	**No. influenza-positive (%)[a]**	**Unadjusted OR (95% CI)**	**Adjusted OR (95% CI)**
**All ages (≥6 months)**												
14-41	6353	36 (1)	Ref.	Ref.	6536	219 (3)	Ref.	Ref.	6361	44 (1)	Ref.	Ref.
42-69	9746	136 (1)	0.91 (0.59, 1.41)	1.02 (0.66, 1.58)	10650	1040 (10)	1.04 (0.84, 1.28)	1.06 (0.86, 1.31)	9825	215 (2)	0.77 (0.50, 1.17)	0.78 (0.51, 1.20)
70-97	10933	196 (2)	1.14 (0.71, 1.84)	1.35 (0.84, 2.19)	11876	1139 (10)	1.06 (0.83, 1.34)	1.09 (0.86, 1.40)	11152	415 (4)	0.79 (0.50, 1.26)	0.83 (0.52, 1.32)
98-125	8930	168 (2)	1.11 (0.65, 1.89)	1.39 (0.81, 2.39)	9442	680 (7)	1.23 (0.93, 1.63)	1.29 (0.97, 1.71)	9328	566 (6)	0.89 (0.55, 1.45)	0.97 (0.59, 1.58)
126-153	6387	110 (2)	1.23 (0.68, 2.21)	1.63 (0.90, 2.98)	6611	334 (5)	1.37 (0.97, 1.92)	1.44 (1.02, 2.04)	6742	465 (7)	0.79 (0.47, 1.31)	0.89 (0.53, 1.49)
≥154[b]	1115	17 (2)	1.93 (0.86, 4.36)**	2.59 (1.13, 5.91)*	1134	36 (3)	1.21 (0.72, 2.02)**	1.34 (0.79, 2.25)*	1183	85 (7)	0.68 (0.39, 1.21)**	0.78 (0.44, 1.39)*
Per 28 days[c]			1.11 (0.96, 1.28)††	1.20 (1.03, 1.39) ††			1.12 (1.02, 1.23) ††	1.15 (1.04, 1.26) ††			0.95 (0.86, 1.05)†	1.00 (0.90, 1.11) †
**<18 years**												
14-41	403	≤5	Ref.	Ref.	412	10 (2)	Ref.	Ref.	406	≤5	Ref.	Ref.
42-69	377	≤5	NE	NE	392	19 (5)	0.11 (0.01, 0.94)	0.14 (0.02, 1.22)	382	9 (2)	NE	NE
70-97	376	9 (2)	NE	NE	390	23 (6)	0.09 (0.01, 0.98)	0.16 (0.01, 1.94)	393	26 (7)	NE	NE
98-125	309	≤5	NE	NE	319	13 (4)	0.09 (0.01, 1.29)	0.23 (0.01, 4.14)	332	26 (8)	NE	NE
126-153	165	7 (4)	NE	NE	162	≤5	NE	NE	182	24 (13)	NE	NE
≥154[b]	25	≤5	NE**	NE**	25	≤5	NE**	NE**	25	≤5	NE**	NE**
Per 28 days[c]			0.66 (0.30, 1.45)††	0.64 (0.26, 1.58)††			0.64 (0.31, 1.33)††	0.76 (0.36, 1.65) ††			0.82 (0.55, 1.24) ††	0.88 (0.57, 1.35) ††
**18-64 years**												
14-41	1397	8-12 (1)	Ref.	Ref.	1428	43 (3)	Ref.	Ref.	1392	6-10 (1)	Ref.	Ref.
42-69	1952	38-42 (2)	0.63 (0.28, 1.44)	0.70 (0.31, 1.62)	2069	156 (8)	0.61 (0.36, 1.03)	0.68 (0.39, 1.18)	1955	42 (2)	0.99 (0.30, 3.24)	1.07 (0.31, 3.64)
70-97	2194	79 (4)	1.04 (0.44, 2.47)	1.28 (0.53, 3.10)	2270	155 (7)	0.43 (0.23, 0.80)	0.49 (0.26, 0.93)	2190	75 (3)	0.72 (0.20, 2.58)	0.77 (0.21, 2.85)
98-125	1843	72-76 (4)	1.08 (0.42, 2.79)	1.32 (0.50, 3.47)	1883	114 (6)	0.77 (0.37, 1.58)	1.01 (0.47, 2.14)	1894	125 (7)	0.87 (0.23, 3.26)	0.98 (0.25, 3.82)
126-153	1211	33 (3)	1.29 (0.45, 3.70)	1.57 (0.53, 4.64)	1215	36-40 (3)	0.89 (0.34, 2.32)	1.24 (0.46, 3.36)	1274	96 (8)	0.74 (0.19, 2.92)	0.89 (0.22, 3.67)

≥154[b]	221	5-9 (2-4)	3.87 (0.93, 16.17)**	5.09 (1.18, 22.05)*	215	≤5	NE**	NE*	225	9-13 (4-6)	0.39 (0.08, 1.76) **	0.45 (0.09, 2.13)*
Per 28 days[c]			1.21 (0.94, 1.56)†	1.26 (0.97, 1.64) ††			0.93 (0.72, 1.20)†	0.99 (0.76, 1.30)†			0.88 (0.69, 1.13)††	0.94 (0.73, 1.21)††
**≥65 years**												
14-41	4553	23 (1)	Ref.	Ref.	4696	166 (4)	Ref.	Ref.	4563	33 (1)	Ref.	Ref.
42-69	7417	93 (1)	0.95 (0.55, 1.63)	1.03 (0.60, 1.78)	8189	865 (11)	1.13 (0.89, 1.44)	1.15 (0.90, 1.47)	7488	164 (2)	0.78 (0.48, 1.26)	0.77 (0.48, 1.25)
70-97	8363	108 (1)	1.07 (0.58, 1.96)	1.22 (0.66, 2.24)	9216	961 (10)	1.22 (0.93, 1.60)	1.28 (0.97, 1.68)	8569	314 (4)	0.90 (0.53, 1.52)	0.91 (0.54, 1.55)
98-125	6778	91 (1)	1.09 (0.54, 2.18)	1.27 (0.63, 2.57)	7240	553 (8)	1.33 (0.97, 1.84)	1.41 (1.02, 1.94)	7102	415 (6)	1.08 (0.61, 1.90)	1.09 (0.62, 1.92)
126-153	5011	70 (1)	1.19 (0.55, 2.57)	1.40 (0.65, 3.04)	5234	291-299 (6)	1.43 (0.98, 2.10)	1.55 (1.05, 2.28)	5286	345 (7)	0.98 (0.54, 1.77)	1.00 (0.55, 1.81)
≥154[b]	869	7 (1)	1.05 (0.32, 3.39)**	1.24 (0.38, 4.07)*	894	26-34 (3-4)	1.41 (0.80, 2.49)**	1.56 (0.88, 2.76)*	933	71 (8)	1.03 (0.53, 1.97)**	1.04 (0.54, 2.02)*
Per 28 days[c]			1.11 (0.91, 1.35)††	1.15 (0.94, 1.41)††			1.16 (1.04, 1.29)††	1.20 (1.08, 1.33)††			1.03 (0.91, 1.17)††	1.04 (0.92, 1.19)††

[a] Counts with ≤5 vaccinated cases were suppressed and estimates were replaced with NE (not estimable). Estimates where the reference group (i.e., 14-41 days) had ≤5 vaccinated cases were replaced with NE. Ranges are presented to avoid back-calculation of counts ≤5.

[b] Cochran-Armitage trend tests were conducted to determine the association between time since vaccination group (a categorical variable modelled as a continuous variable) and laboratory-confirmed influenza. P-values for trend tests were marked on the estimate for the last interval (≥154 days); *p <0.05; ** p≥0.05. A P-value <0.05 implies a trend between time since vaccination categories and laboratory-confirmed influenza.

[c] Time since vaccination per 28 days were modelled using restricted cubic splines and its regression coefficients were used to test for linearity. P-values for linearity tests were marked on the calculated estimate; †p <0.05; ††p≥0.05. A P-value ≥0.05 implies that time since vaccination per 28 days is linearly associated with the logit of the outcome (i.e., accepting the null hypothesis that the restricted cubic spline coefficients are zero).

Supplementary Table S6. Odds ratios (OR) by time since vaccination (TSV) against laboratory-confirmed influenza infection of the predominant circulating strain(s) in community-dwelling individuals aged ≥6 months in Ontario, Canada by influenza season.

	Restricted to vaccinated individuals tested between October 1st and March 31st of the following year				Restricted to vaccinated individuals tested during influenza activity			
**TSV (days)**	**No. vaccinated**	**No. influenza-positive (%)[a]**	**Unadjusted OR (95% CI)**	**Adjusted OR (95% CI)**	**No. vaccinated**	**No. influenza-positive (%)[a]**	**Unadjusted OR (95% CI)**	**Adjusted OR (95% CI)**
**2020-2011 - Influenza A/H3N2**								
	October 1st, 2010 – March 31st, 2011				*November 14th, 2010 – April 9th, 2011*			
14-41	206	9 (4)	Ref.	Ref.	172	9 (5)	Ref.	Ref.
42-69	446	107 (24)	2.19 (0.80, 6.03)	2.44 (0.83, 7.12)	448	107 (24)	2.16 (0.78, 5.96)	2.43 (0.83, 7.12)
70-97	623	103 (17)	1.80 (0.60, 5.40)	1.96 (0.61, 6.31)	625	103 (16)	1.75 (0.58, 5.25)	1.91 (0.59, 6.14)
98-125	430	27 (6)	1.23 (0.34, 4.45)	1.47 (0.38, 5.71)	430	27 (6)	1.20 (0.33, 4.33)	1.43 (0.37, 5.55)
126-153	329	13 (4)	1.66 (0.31, 8.93)	1.48 (0.25, 8.80)	364	13 (4)	1.62 (0.30, 8.68)	1.42 (0.24, 8.48)
≥154[b]	46	0	0**	0*	109	≤5	NE**	NE*
Per 28 days[c]			0.90 (0.60, 1.34)††	0.87 (0.57, 1.34)††			0.87 (0.59, 1.30)††	0.84 (0.55, 1.29) ††
**2011-2012 – Influenza B**								
	October 1st, 2011 – March 31st, 2012				*January 29th, 2012 – May 19th, 2012*			
14-41	381	≤5	Ref.	Ref.	13	≤5	Ref.	Ref.
42-69	509	≤5	0.30 (0.06, 1.42)	0.35 (0.07, 1.71)	54	≤5	NE	NE
70-97	496	9 (2)	0.24 (0.06, 0.97)	0.29 (0.07, 1.26)	191	8 (4)	0.41 (0.04, 4.15)	0.37 (0.04, 3.86)
98-125	498	40 (8)	0.39 (0.10, 1.61)	0.56 (0.12, 2.49)	506	39 (8)	0.52 (0.05, 4.90)	0.56 (0.06, 5.47)
126-153	400	45 (11)	0.33 (0.08, 1.40)	0.50 (0.11, 2.31)	545	53 (10)	0.45 (0.05, 4.32)	0.51 (0.05, 5.06)
≥154[b]	123	13 (11)	0.32 (0.07, 1.51)**	0.45 (0.09, 2.37)*	762	51 (7)	0.53 (0.05, 5.30)**	0.62 (0.06, 6.40)*
Per 28 days[c]			0.97 (0.74, 1.27)††	1.05 (0.79, 1.40)††			1.04 (0.82, 1.32)††	1.13 (0.88, 1.46)††
**2012-2013 – Influenza A/H3N2§**								
	October 1st, 2012 – March 31st, 2013				*November 4th, 2012 – May 11th, 2013*			
14-41	227	8 (4)	Ref.	Ref.	204	7 (3)	Ref.	Ref.
42-69	592	115 (19)	1.81 (0.63, 5.21)	1.82 (0.62, 5.29)	594	115 (19)	1.78 (0.62, 5.13)	1.78 (0.61, 5.17)
70-97	785	142 (18)	1.72 (0.55, 5.33)	1.83 (0.58, 5.73)	785	142 (18)	1.69 (0.55, 5.25)	1.79 (0.57, 5.61)
98-125	488	33 (7)	1.70 (0.48, 5.99)	1.82 (0.51, 6.51)	488	33 (7)	1.68 (0.48, 5.90)	1.78 (0.50, 6.37)
126-153	366	10-14 (3-4)	15.03 (2.52, 89.78)	15.52 (2.55, 94.46)	371	14 (4)	14.80 (2.48, 88.42)	15.17 (2.49, 92.28)
≥154[b]	104	≤5	NE**	NE**	523	≤5	NE**	NE**

Per 28 days[c]			1.39 (0.93, 2.06)[†]	1.49 (1.00, 2.23)[†]			1.37 (0.92, 2.03)[†]	1.47 (0.99, 2.20)[†]
**2013-2014 – Influenza A/H1N1**								
		October 1st, 2013 – March 31st, 2014				*December 1st, 2013 – May 24th, 2014*		
14-41	586	12 (2)	Ref.	Ref.	290	12 (4)	Ref.	Ref.
42-69	991	65 (7)	1.35 (0.67, 2.75)	1.56 (0.75, 3.25)	957	65 (7)	1.35 (0.66, 2.74)	1.55 (0.75, 3.23)
70-97	1161	69 (6)	1.72 (0.80, 3.73)	2.13 (0.96, 4.72)	1161	69 (6)	1.71 (0.79, 3.69)	2.10 (0.95, 4.67)
98-125	828	17 (2)	1.67 (0.60, 4.64)	2.30 (0.80, 6.61)	837	17 (2)	1.65 (0.59, 4.60)	2.27 (0.79, 6.56)
126-153	654	5-9 (1)	5.56 (1.29, 23.84)	7.88 (1.78, 34.92)	808	10 (1)	5.67 (1.32, 24.24)	8.02 (1.81, 35.42)
≥154[b]	148	≤5	NE**	NE*	1392	≤5	NE**	NE*
Per 28 days[c]			1.38 (1.00, 1.90)[††]	1.49 (1.07, 2.09)[††]			1.34 (0.97, 1.85)[††]	1.46 (1.05, 2.03)[††]
**2013-2014 – Influenza B**								
		October 1st, 2013 – March 31st, 2014				*December 1st, 2013 – May 24th, 2014*		
14-41	574	0	Ref.	Ref.	278	0	Ref.	Ref.
42-69	930	≤5	NE	NE	896	≤5	NE	NE
70-97	1104	11-15 (1)	NE	NE	1104	12 (1)	NE	NE
98-125	851	40 (5)	NE	NE	860	40 (5)	NE	NE
126-153	734	89 (12)	NE	NE	925	127 (14)	NE	NE
≥154[b]	171	24 (14)	NE**	NE*	1608	218 (14)	NE**	NE*
Per 28 days[c]			1.05 (0.74, 1.48)[††]	1.16 (0.81, 1.66)[††]			1.00 (0.80, 1.25)[††]	1.01 (0.80, 1.27)[††]
**2014-2015 – Influenza A/H3N2§**								
		October 1st, 2014 – March 31st, 2015				*November 23rd, 2014 – May 16th, 2015*		
14-41	888	100 (11)	Ref.	Ref.	633	91 (14)	Ref.	Ref.
42-69	1699	325 (19)	1.03 (0.75, 1.41)	1.04 (0.75, 1.43)	1701	324 (19)	1.01 (0.74, 1.38)	1.02 (0.74, 1.41)
70-97	1816	285 (16)	1.00 (0.69, 1.46)	1.01 (0.69, 1.49)	1826	285 (16)	0.98 (0.67, 1.42)	1.00 (0.68, 1.47)
98-125	1202	102 (8)	1.01 (0.62, 1.65)	1.04 (0.63, 1.73)	1202	102 (8)	0.98 (0.60, 1.61)	1.03 (0.62, 1.71)
126-153	794	39-43 (5)	1.45 (0.72, 2.92)	1.52 (0.74, 3.10)	943	46 (5)	1.41 (0.70, 2.83)	1.50 (0.74, 3.07)
≥154[b]	153	≤5	NE**	NE*	1162	18 (2)	1.04 (0.34, 3.21)**	1.06 (0.34, 3.32)*
Per 28 days[c]			1.08 (0.91, 1.28)[††]	1.09 (0.91, 1.31)[††]			1.04 (0.87, 1.23)[††]	1.05 (0.88, 1.26)[††]
**2015-2016 – Influenza A/H1N1**								
		October 1st, 2015 – March 31st, 2016				*January 17th, 2016 – May 28th, 2016*		
14-41	970	6 (1)	Ref.	Ref.	70	≤5	Ref.	Ref.
42-69	1183	10 (1)	0.36 (0.12, 1.08)	0.44 (0.14, 1.37)	312	9 (3)	0.75 (0.15, 3.66)	1.01 (0.20, 5.18)
70-97	1295	49 (4)	0.53 (0.19, 1.46)	0.74 (0.26, 2.14)	1079	47 (4)	0.90 (0.21, 3.96)	1.38 (0.30, 6.31)
98-125	1498	115 (8)	0.65 (0.23, 1.81)	0.93 (0.32, 2.74)	1641	120 (7)	1.14 (0.26, 5.03)	1.79 (0.39, 8.26)

126-153	1065	76 (7)	0.72 (0.25, 2.09)	1.13 (0.37, 3.48)	1525	84 (6)	1.14 (0.25, 5.17)	1.95 (0.41, 9.34)
≥154[b]	138	6 (4)	0.67 (0.17, 2.67)[**]	1.07 (0.25, 4.54)[*]	1687	26 (2)	0.95 (0.19, 4.77)[**]	1.70 (0.32, 9.00)[*]
Per 28 days[c]			1.11 (0.91, 1.36)[††]	1.21 (0.98, 1.49)[††]			1.10 (0.91, 1.32)[††]	1.20 (0.98, 1.46)[††]
**2015-2016 – Influenza B**								
		October 1st, 2015 – March 31st, 2016				*January 17th, 2016 – May 28th, 2016*		
14-41	968	≤5	Ref.	Ref.	69	≤5	Ref.	Ref.
42-69	1185	11-15 (1)	0.73 (0.21, 2.58)	0.95 (0.27, 3.40)	312	9 (3)	1.20 (0.14, 10.09)	1.65 (0.19, 14.22)
70-97	1270	24 (2)	0.44 (0.12, 1.63)	0.61 (0.16, 2.30)	1058	26 (2)	0.75 (0.09, 5.94)	1.13 (0.14, 9.23)
98-125	1472	89 (6)	0.59 (0.16, 2.21)	1.04 (0.27, 3.99)	1616	95 (6)	0.89 (0.11, 7.18)	1.54 (0.19, 12.83)
126-153	1065	76 (7)	0.39 (0.10, 1.48)	0.80 (0.20, 3.18)	1560	119 (8)	0.67 (0.08, 5.42)	1.27 (0.15, 10.71)
≥154[b]	138	6 (4)	0.17 (0.03, 0.82)[*]	0.34 (0.07, 1.73)[*]	1769	108 (6)	0.44 (0.05, 3.67)[*]	0.95 (0.11, 8.19)[*]
Per 28 days[c]			0.75 (0.61, 0.91)[†]	0.90 (0.73, 1.11)[†]			0.78 (0.67, 0.91)[††]	0.90 (0.76, 1.06)[††]
**2016-2017 – Influenza A/H3N2**								
		October 1st, 2016 – March 31st, 2017				*November 20th, 2016 – May 27th, 2017*		
14-41	858	59 (7)	Ref.	Ref.	709	56 (8)	Ref.	Ref.
42-69	1809	334 (18)	0.87 (0.57, 1.32)	0.91 (0.59, 1.40)	1805	334 (19)	0.85 (0.55, 1.30)	0.89 (0.58, 1.38)
70-97	1921	339 (18)	0.90 (0.56, 1.46)	0.98 (0.60, 1.61)	1923	339 (18)	0.87 (0.54, 1.41)	0.96 (0.59, 1.57)
98-125	1347	213 (16)	1.04 (0.59, 1.84)	1.16 (0.65, 2.08)	1349	213 (16)	1.00 (0.56, 1.78)	1.13 (0.63, 2.02)
126-153	907	90 (10)	0.98 (0.49, 1.95)	1.14 (0.56, 2.30)	1055	100 (9)	0.94 (0.47, 1.88)	1.10 (0.54, 2.23)
≥154[b]	99	8 (8)	0.75 (0.25, 2.20)[**]	0.94 (0.32, 2.82)[*]	1418	54 (4)	0.76 (0.30, 1.88)[**]	0.97 (0.39, 2.45)[*]
Per 28 days[c]			1.07 (0.88, 1.31)[††]	1.14 (0.93, 1.40)[††]			1.05 (0.87, 1.27)[††]	1.12 (0.92, 1.37)[††]
**2017-2018 – Influenza A/H3N2[§]**								
		October 1st, 2017 – March 31st, 2018				*November 26th, 2017 – May 12th, 2018*		
14-41	1172	28 (2)	Ref.	Ref.	732	23 (3)	Ref.	Ref.
42-69	1834	120 (7)	1.00 (0.57, 1.74)	1.01 (0.58, 1.77)	1846	120 (7)	0.99 (0.56, 1.75)	1.00 (0.57, 1.76)
70-97	2139	206 (10)	1.11 (0.59, 2.07)	1.13 (0.60, 2.12)	2150	206 (10)	1.09 (0.58, 2.05)	1.11 (0.59, 2.09)
98-125	1901	223 (12)	1.60 (0.81, 3.16)	1.62 (0.82, 3.22)	1910	223 (12)	1.55 (0.78, 3.08)	1.56 (0.78, 3.11)
126-153	1185	97 (8)	1.70 (0.80, 3.64)	1.70 (0.79, 3.68)	1445	110 (8)	1.64 (0.76, 3.51)	1.61 (0.74, 3.49)
≥154[b]	176	11 (6)	1.69 (0.60, 4.77)[*]	1.79 (0.62, 5.15)[*]	1297	52 (4)	1.80 (0.73, 4.44)[*]	1.82 (0.73, 4.55)[*]
Per 28 days[c]			1.26 (1.04, 1.54)[††]	1.27 (1.03, 1.55)[††]			1.22 (1.01, 1.48)[††]	1.21 (0.99, 1.47)[††]
**2017-2018 – Influenza B[§]**								
		October 1st, 2017 – March 31st, 2018				*November 26th, 2017 – May 12th, 2018*		
14-41	1173	29 (2)	Ref.	Ref.	734	25 (3)	Ref.	Ref.
42-69	1891	177 (9)	0.84 (0.51, 1.41)	0.84 (0.50, 1.40)	1903	177 (9)	0.83 (0.49, 1.39)	0.83 (0.49, 1.39)

70-97	2284	351 (15)	0.96 (0.55, 1.67)	0.96 (0.55, 1.68)	2295	351 (15)	0.93 (0.54, 1.63)	0.94 (0.54, 1.64)
98-125	2029	351 (17)	1.05 (0.58, 1.90)	1.04 (0.57, 1.89)	2038	351 (17)	1.01 (0.56, 1.84)	1.00 (0.55, 1.83)
126-153	1241	153 (12)	0.96 (0.50, 1.84)	0.96 (0.50, 1.85)	1506	171 (11)	0.92 (0.48, 1.78)	0.92 (0.48, 1.78)
≥154[b]	186	21 (11)	0.99 (0.43, 2.28)**	0.99 (0.42, 2.29)*	1325	80 (6)	1.02 (0.48, 2.18)**	1.00 (0.47, 2.16)*
Per 28 days[c]			1.05 (0.90, 1.23)††	1.07 (0.91, 1.25)††			1.03 (0.88, 1.20)††	1.04 (0.89, 1.21)††
**2018-2019 – Influenza A/H1N1**								
		October 1st, 2018 – March 31st, 2019				*November 25th, 2018 – May 25th, 2019*		
14-41	1271	16 (1)	Ref.	Ref.	885	15 (2)	Ref.	Ref.
42-69	1675	48 (3)	0.77 (0.38, 1.54)	0.93 (0.46, 1.88)	1680	48 (3)	0.77 (0.38, 1.54)	0.94 (0.46, 1.91)
70-97	1761	58 (3)	0.98 (0.44, 2.19)	1.21 (0.53, 2.75)	1771	58 (3)	0.97 (0.43, 2.17)	1.21 (0.53, 2.77)
98-125	1362	22 (2)	0.49 (0.16, 1.47)	0.70 (0.23, 2.15)	1372	22 (2)	0.48 (0.16, 1.45)	0.71 (0.23, 2.17)
126-153	967	3-7 (1)	0.21 (0.05, 0.90)	0.30 (0.07, 1.36)	1242	8 (1)	0.21 (0.05, 0.91)	0.32 (0.07, 1.41)
≥154[b]	157	≤5	NE**	NE*	1934	≤5	NE**	NE*
Per 28 days[c]			0.85 (0.59, 1.24)††	0.96 (0.66, 1.41)††			0.81 (0.56, 1.18)††	0.92 (0.63, 1.35)††

[a] Counts with ≤5 vaccinated cases were suppressed and estimates were replaced with NE (not estimable). Estimates where the reference group (i.e., 14-41 days) had ≤5 vaccinated cases were replaced with NE. Ranges are presented to avoid back-calculation of counts ≤5.

[b] Cochran-Armitage trend tests were conducted to determine the association between time since vaccination group (a categorical variable modelled as a continuous variable) and laboratory-confirmed influenza. P-values for trend tests were marked on the estimate for the last interval (≥154 days); *p <0.05; ** p≥0.05. A P-value <0.05 implies a trend between time since vaccination categories and laboratory-confirmed influenza.

[c] Time since vaccination per 28 days were modelled using restricted cubic splines and its regression coefficients were used to test for linearity. P-values for linearity tests were marked on the calculated estimate; †p <0.05; ††p≥0.05. A P-value ≥0.05 implies that time since vaccination per 28 days is linearly associated with the logit of the outcome (i.e., accepting the null hypothesis that the restricted cubic spline coefficients are zero).

[§] Influenza seasons with known vaccine mismatch (and consequently low influenza vaccine effectiveness) in Canada.
